# Oral Vaccination With a Formulation Combining *Rhipicephalus microplus* Subolesin With Heat Inactivated *Mycobacterium bovis* Reduces Tick Infestations in Cattle

**DOI:** 10.3389/fcimb.2019.00045

**Published:** 2019-03-01

**Authors:** Marinela Contreras, Paul D. Kasaija, Octavio Merino, Ned I. de la Cruz-Hernandez, Christian Gortazar, José de la Fuente

**Affiliations:** ^1^SaBio, Instituto de Investigación en Recursos Cinegéticos IREC-CSIC-UCLM-JCCM, Ciudad Real, Spain; ^2^National Livestock Resources Research Institute (NaLIRRI/NARO), Tororo, Uganda; ^3^Facultad de Medicina Veterinaria y Zootecnia, Universidad Autónoma de Tamaulipas, Ciudad Victoria, Mexico; ^4^Department of Veterinary Pathobiology, Center for Veterinary Health Sciences, Oklahoma State University, Stillwater, OK, United States

**Keywords:** subolesin, cattle tick, vaccine, oral vaccination, *Rhipicephalus microplus*, heat inactivated *Mycobacterium bovis*

## Abstract

Vaccines are an environmentally friendly alternative to acaracides for the control of tick infestations, to reduce the risk for tick-borne diseases affecting human and animal health worldwide, and to improve animal welfare and production. Subolesin (SUB, also known as 4D8) is the functional homolog of Akirin2 involved in the regulation of development and innate immune response, and a proven protective antigen for the control of ectoparasite infestations and pathogen infection. Oral vaccination combining protein antigens with immunostimulants has proven efficacy with increased host welfare and safety, but has not been used for the control of tick infestations. Here we describe the efficacy of oral vaccination with a formulation combining *Rhipicephalus microplus* SUB and heat inactivated *Mycobacterium bovis* (IV) on cattle tick infestations and fertility. The levels of IgG antibody titers against SUB and *M. bovis* P22, and the expression of selected immune response genes were determined and analyzed as possible correlates of protection. We demonstrated that oral immunization with the SUB+IV formulation resulted in 51% reduction in the number of female ticks and 30% reduction in fertility with an overall efficacy of 65% in the control of *R. microplus* infestations by considering the cumulative effect on reducing tick survival and fertility in cattle. The *akr2, IL-1*β, and *C3* mRNA levels together with antibody levels against SUB correlated with vaccine efficacy. The effect of the oral immunization with SUB+IV in cattle on tick survival and fertility is essential to reduce tick infestations, and extended previous results on the effect of *R. microplus* SUB for the control of cattle tick infestations. These results support the development of oral vaccines formulations for the control of tick infestations and the incidence of tick-borne diseases.

## Introduction

Ticks are arthropod vectors of pathogens affecting human and animal health as well as animal welfare and production worldwide (Jongejan and Uilenberg, 2004; de la Fuente et al., 2008; Rashid et al., 2018). The cattle tick *Rhipicephalus microplus* Canestrini (Acari: Ixodidae) are economically important as parasites of a variety of livestock species with an impact on cattle industry in tropical and subtropical regions of the world (Rashid et al., 2018).

Despite the use of traditional cattle tick control methods such as the use of chemical acaricides, habitat management, and genetic selection of animals with higher resistance to ticks, tick prevalence continues to be a major economic problem for the cattle industry (de la Fuente et al., 2017; Rashid et al., 2018). This persistent problem is due to several factors including acaracide resistance in ticks and safety issues associated with these chemicals, which support the development of vaccines as an effective and environmentally sound approach for the control of tick infestations (de la Fuente and Contreras, 2015; de la Fuente et al., 2016b; 2017; de la Fuente, 2018). The commercial vaccines based on the *R. microplus* BM86 or BM95 recombinant antigens proved their efficacy for the control of cattle tick infestations and the reduction in the prevalence of certain tick-borne pathogens (de la Fuente et al., 2007, 2017; de la Fuente and Contreras, 2015; Rodríguez-Mallon, 2016; de la Fuente, 2018).

Tick Subolesin (SUB, also known as 4D8) is the functional ortholog of Akirin2 and is involved in the regulation of different biological processes including development and innate immune response (Artigas-Jerónimo et al., 2018). SUB was discovered as a tick protective antigen (Almazán et al., 2010), and since then it has shown vaccination efficacy for the control of infestations by different arthropod ectoparasite species and pathogen infection and transmission (recently reviewed by de la Fuente and Contreras, 2015; Artigas-Jerónimo et al., 2018).

Recent advances in tick vaccine research have resulted in the identification of new protective antigens for the control of tick infestations (recently reviewed by de la Fuente and Contreras, 2015; de la Fuente et al., 2016b, 2017; de la Fuente, 2018). However, research aimed at improving tick vaccine efficacy and safety by combining protective antigens and oral formulations is still to be done. Oral or intranasal vaccine formulations are easier to administer, and have proven efficacy with increased host welfare and safety by reducing stress and the risk of contamination or infection at the injection site and pathogen mechanical transmission (Wang et al., 2015; Lawan et al., 2018). However, orally delivered protein vaccines have a relatively low immunogenicity and antigen stability after immunization that require vaccine formulations with selected combinations of antigens and immunostimulants, and needleless delivery systems (Fry et al., 2012; Wang et al., 2015). In this context, the heat inactivated *Mycobacterium bovis* (IV) has been shown to activate the innate immune response-mediated trained immunity through complement component 3 (C3) to reduce mycobacterial infection and tuberculosis-like lesions in cattle, deer, pig, and zebrafish orally or systemically vaccinated with IV (Beltrán-Beck et al., 2014; de la Fuente et al., 2016a; Juste et al., 2016; Thomas et al., 2017; López et al., 2018, 2019; Risalde et al., 2018). Therefore, IV appears as a good immunostimulant candidate for oral vaccine formulations (de la Fuente et al., 2016a).

As a proof of concept of oral tick vaccine formulations, in this study we orally vaccinated cattle via needleless syringe using a formulation combining *R. microplus* SUB with IV for the control of cattle tick infestation. The results showed an effect of the oral vaccination on the reduction in the number of female ticks and fertility. Additionally, the *akirin2* (*akr2*), *interleukin-1beta* (*IL-1*β), and *C3* mRNA levels together with antibody levels against SUB correlated with vaccine efficacy. These results support research for the development of oral vaccines formulations for the control of tick infestations and the incidence of tick-borne diseases.

## Materials and Methods

### Ticks

The *R. microplus* (Susceptible Media Joya strain, CENAPA, Mexico) ticks were obtained from a laboratory colony maintained at the University of Tamaulipas, Mexico (Merino et al., 2011). Tick larvae were fed on cross-bred *Bos taurus* cattle and collected after repletion to allow for oviposition and hatching in humidity chambers at 12 h light:12 h dark photoperiod, 22–25°C and 95% relative humidity (RH). Larvae were used for infestations at 15 days after hatching from eggs.

### Antigen Production and Vaccine Formulations

**Figure 1:**
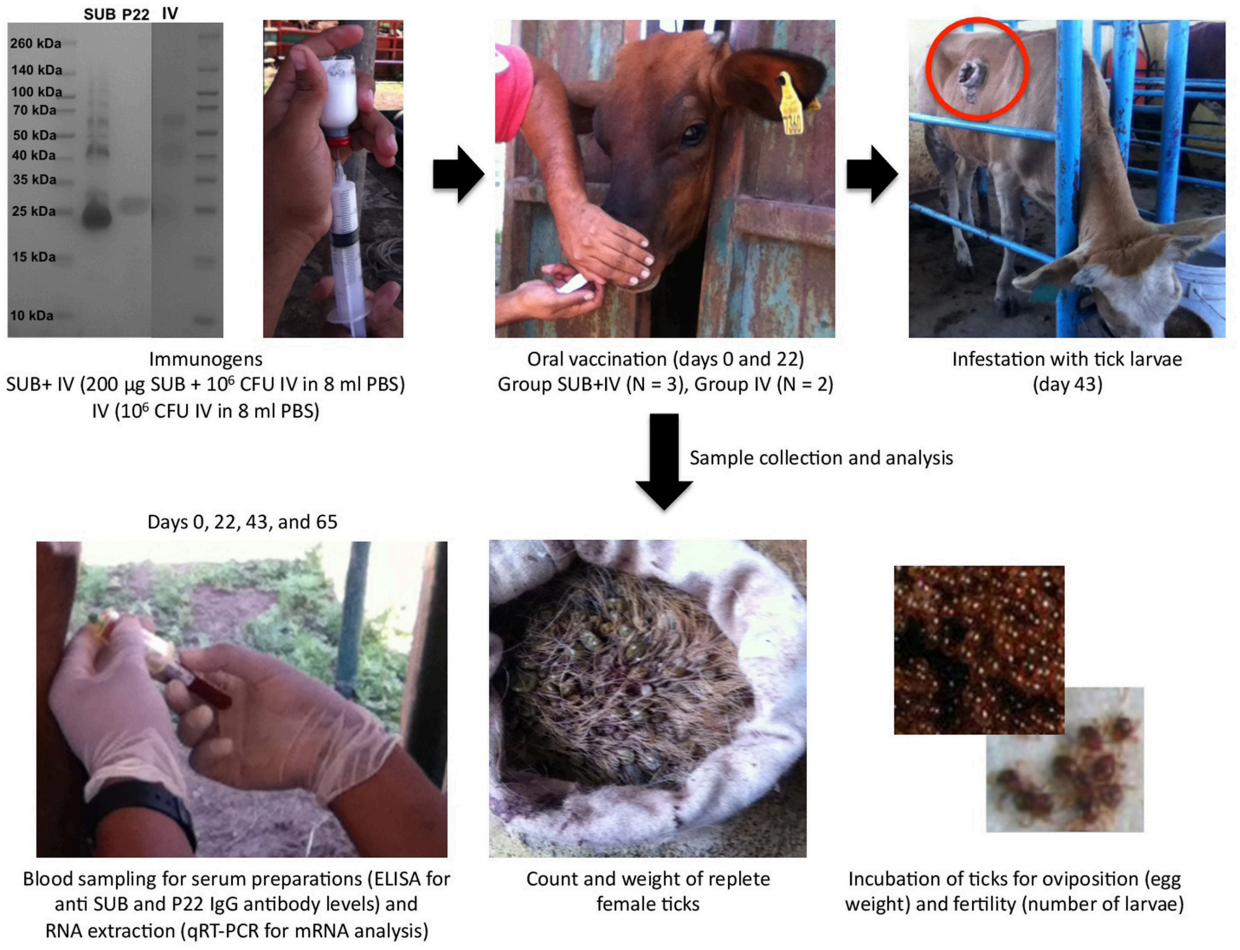
Experimental design. The antigens used in the study included recombinant *R. microplus* SUB (>95% purity), heat inactivated *M. bovis* IV proteins and *M. bovis* P22 (as an example, a Coomassie-blue stained SDS-12% polyacrilamide gel is shown with the three antigens and spectra multicolor molecular weight markers from Thermo Scientific). Cattle were orally vaccinated via needleless syringe on days 0 and 22 using a formulation combining *R. microplus* SUB antigen with IV as immunostimulator (SUB+IV group) or IV alone (IV group) for comparative analyses between both groups. After vaccination, cattle were infested with *R. microplus* larvae on day 43 for the analysis of vaccine efficacy on the control of tick development and fertility, and the identification of possible correlates of protection and immune mechanisms based on the host antibody response and the mRNA levels for selected immune response genes.

The synthetic *R. microplus* histidine-tag recombinant SUB (Genbank accession number GQ456170) with optimized codon usage for *Escherichia coli* was produced in *E. coli* BL21 and purified to >95% purity (Figure 1) by Ni affinity chromatography using 1 ml HisTrap FF columns mounted on an AKTA–FPLC system (GE Healthcare, Piscataway, NJ, USA) in the presence of 7 M urea lysis buffer as previously described (Almazán et al., 2010; Contreras et al., 2015). Protein concentration was measured using the Pierce® BCA protein assay kit (Thermo Scientific, Rockford, IL, USA).

The IV was prepared from *M. bovis* field isolate Neiker 1403 (spoligotype SB0339 originally isolated from a naturally infected wild boar) IV at Neiker-Tecnalia (Derio, Spain) under good manufacturing practices as previously described (Beltrán-Beck et al., 2014; Juste et al., 2016; Risalde et al., 2018). Briefly, the isolate was cultivated for 2–3 weeks in Middlebrook 7H9 medium enriched with OADC growth supplement (Sigma-Aldrich, St. Louis, MI, USA), and inactivated in a shaking water bath at 81–83°C for 40 min. The inactivated inoculum was cultured in BACTEC Mycobacterial Growth Indicator Tubes (Becton Dickinson; Franklin Lakes, NJ, USA) and onto OADC agar solidified 7H9 plates in triplicate (100 μl each) to confirm the absence of viable mycobacteria. The final *M. bovis* IV preparation contained approximately the equivalent of 10^7^ colony forming units (cfu) in 0.2 ml of PBS.

For vaccine formulation, purified recombinant SUB protein (200 μg) was mixed with 6 x 10^6^ cfu IV in 18 ml PBS for SUB+IV group, or the same amount of IV was placed in 18 ml PBS for control IV group. Vaccine formulations were stored at 4°C until used.

### Cattle Vaccination and Tick Infestation

Three and two 10-month-old European crossbred calves were randomly assigned to SUB+IV and IV vaccinated groups. Vaccines were administered via needleless syringe in the lateral region of the mouth followed by slightly raising the head of the calves (Jones et al., 2016). Cattle were vaccinated at days 0 and 22, and then infested at day 43 with 500 *R. microplus* larvae in single cells glued on the back of the calves. Adult engorged female ticks dropping from cattle were daily collected, counted, and weighted. All the collected adult female ticks were assessed for oviposition (egg mass weight/tick) and egg fertility (number of larvae/tick) to calculate vaccine efficacy (E) as previously reported (Merino et al., 2011, 2013) using only parameters with significant differences between groups (Table 1). The personnel vaccinating cattle and collecting the ticks were “blinded” as to which group animals belonged. Data were analyzed statistically to compare results between individuals fed on SUB+IV and IV vaccinated calves by Student's *t*-test with unequal variance (*p* = 0.05).

**Table 1:** Results of the oral vaccination on cattle tick infestations.

**Group (cattle no.)**	**No. female ticks**	**Female tick weight (mg)**	**Oviposition egg mass/tick (mg)**	**Fertility (No. larvae/tick)**
SUB+IV (1)	36	233 ± 92	98 ± 61	592 ± 490
SUB+IV (3)	59	210 ± 68	116 ± 37	961 ± 620
SUB+IV (5)	73	234 ± 47	104 ± 38	819 ± 610
Average ± S.D.	56 ± 19^*^	226 ± 14	106 ± 9	791 ± 186^**^
Control IV (2)	113	206 ± 53	114 ± 40	1, 118 ± 571
Control IV (4)	116	207 ± 54	114 ± 39	1, 157 ± 558
Average ± S.D.	115 ± 2	207 ± 1	114 ± 0	1, 138 ± 28

Results are shown for each vaccinated and infested cattle (N = 3 for SUB+IV and N = 2 for IV). Data was analyzed statistically to compare results between ticks fed on SUB+IV and IV vaccinated cattle (

* p = 0.02

** *p = 0.04; Student's t-test with unequal variance). Vaccine efficacy (E = 65%) was calculated as E = 100 x [1-(DT x DF)], where DT = No. female ticks in SUB+IV vaccinated calves/female ticks in IV vaccinated calves and DF = No. larvae/tick in SUB+IV vaccinated calves/No. larvae/tick in IV vaccinated calves*.

### Analysis of Cattle IgG Antibody Response by ELISA

Serum samples were prepared from blood samples collected from each calf before each immunization (days 0 and 22), tick infestation (day 43) and at the end of the experiment (day 65), and stored at −20°C until analysis. An indirect ELISA test was performed to detect IgG antibodies against *R. microplus* SUB and *M. bovis* P22 proteins as described previously (Merino et al., 2011, 2013; Infantes-Lorenzo et al., 2018). High absorption capacity polystyrene microtiter plates were coated with 100 μl (0.01 μg/ml solution of purified recombinant proteins) per well in carbonate-bicarbonate buffer (Sigma-Aldrich). After an overnight incubation at 4°C, coated plates were blocked with 100 μl/well of blocking solution (5% skim milk in PBS). Serum samples or PBS as negative control were diluted (1:1000, 1:5000 and 1:10000, v/v) in blocking solution and 100 μl/well were added into duplicate wells of the antigen-coated plates. After an overnight incubation at 4°C, the plates were washed three times with a washing solution (PBS containing 0.05% Tween 20) and then incubated with 1:20,000 rabbit anti-bovine immunoglubolin G (IgG)–horseradish peroxidase conjugate (Sigma-Aldrich) for 1 h at 37°C. After three washes with washing solution, 100 μl/well of substrate solution (Fast OPD, Sigma-Aldrich) was added. Finally, the reaction was stopped with 50 μl/well of 2 N H_2_SO_4_ and the optical density (OD) was measured in a spectrophotometer at 450 nm. Antibody titers in SUB+IV and IV vaccinated cattle were expressed as the O.D_450nm_ (O.D_cattlesera_ – O.D_PBScontrol_) and compared between groups by ANOVA test (*p* = 0.05; https://www.socscistatistics.com/tests/anova/Default2.aspx).

### Analysis of Gene Expression by qRT-PCR

Total RNA was extracted from blood samples collected from each calf before each immunization (days 0 and 22), tick infestation (day 43) and at the end of the experiment (day 65) using TRI Reagent BD (Sigma-Aldrich, St. Louis, MI, USA) following the manufacturer's instructions. Gene expression profiles from selected genes involved in immunity *C3* (NM_001040469.2), tumor necrosis factor alpha (*TNF-*α; AF348421)*, IL-1*β (NM174093), interleukin 2 (*IL-2*; NM180997), *akr2* (NM_001110087.1), and interleukin 12 (*IL-12*; U11815.1) were analyzed by qRT-PCR using the following gene-specific forward (F) and reverse (R) 5′- 3′ primers and annealing temperature (*C3*, F: ATTGCCAGGTTCTTGTACGG and R: GTCACTGCCTGATTGCAAGA, 56°C; *TNF-*α, F: CCTCACCCACACCATCAG, and R: GCGATCTCCCTTCTCCAG, 54°C; *IL-1*β, F: TCAGAATGGAAACCCTCTCTC and R: GCATGGATCAGACAACAGTG, 56°C; *IL-12*, F: AAGTGAAGTCATTGCTGCTG and R: TGTCCATTGAATCCTTGATCTC, 54°C; *akr2*, F: CATTTATGGGCTGCCTTGTT and R: TGCACAGCTTCTACCACGAC, 54°C; *IL-12*, F: TCTGGACACTTCACCTGCTG and R: TGCACAGCTTCTACCACGAC, 58°C) and a Quantitect SYBR Green RT-PCR Kit in a Rotor Gene Q thermocycler (Qiagen, Inc. Valencia, CA, USA) following manufacturer's recommendations. A dissociation curve was run at the end of the reaction to ensure that only one amplicon was formed and that the amplicon denatured consistently in the same temperature range for every sample (Ririe et al., 1997). The mRNA Ct values were normalized against *Bos taurus ß-actin* (AY141970.1, F: GGCCGAGCGGAAATCG and R: GCCATCTCCTGCTCGAAGTC, 52°C) using the genNorm ddCT method (Livak and Schmittgen, 2001). The normalized mRNA levels (mean of duplicated and normalized Ct values) were compared between SUB+IV and IV vaccinated groups by ANOVA test (*p* = 0.05; https://www.socscistatistics.com/tests/anova/Default2.aspx).

### Correlation Analysis

Two different Spearman's Rho correlation analyses (*p* = 0.05; https://www.socscistatistics.com/tests/spearman/Default2.aspx) were conducted for the identification of putative correlates of vaccine efficacy. The analysis was conducted in individual SUB+IV and IV vaccinated cattle. The first analysis was conducted between the number of female ticks or larvae/tick and antibody titers (O.D_450nm_) or the normalized mRNA levels at the time of tick infestations (day 43) and at the end of the experiment (day 65). The second analysis was conducted between the normalized mRNA levels and antibody titers (O.D_450nm_) at the time of tick infestations (day 43) and the end of the experiment (day 65).

## Results and Discussion

### Rationale and Experimental Design

Despite the proven efficacy of *R. microplus* BM86 and BM95 based vaccines for the control of cattle tick infestations (de la Fuente et al., 2007, 2017; de la Fuente and Contreras, 2015; Rodríguez-Mallon, 2016; de la Fuente, 2018) and recent advances in the identification of new tick protective antigens (de la Fuente and Contreras, 2015; de la Fuente et al., 2016b, 2017; de la Fuente, 2018), research is needed for the development of vaccine formulations with higher efficacy and safety for the control of tick infestations and tick-borne diseases (de la Fuente, 2018). To address this challenge, the objective of this study was to provide a proof of concept for oral vaccine formulations for the control of cattle tick infestations, and the identification of candidate correlates of vaccine efficacy. Oral vaccine formulations are easier to administer and offer the possibility of reducing stress and vaccination-associated risks while increasing protective efficacy with effective immunostimulants (Wang et al., 2015; Lawan et al., 2018).

The experimental design for this study included cattle vaccination and *R. microplus* infestations for the analysis of vaccine efficacy on the control of tick development and fertility (Figure 1). Cattle were orally vaccinated via needleless syringe using a formulation combining *R. microplus* SUB antigen with proven efficacy in the control of ectoparasite infestations (de la Fuente and Contreras, 2015; Artigas-Jerónimo et al., 2018) and IV with immunostimulatory activity (Beltrán-Beck et al., 2014; de la Fuente et al., 2016a; Juste et al., 2016; Thomas et al., 2017; López et al., 2018, 2019; Risalde et al., 2018) (SUB+IV group) or IV alone (IV group) for comparative analyses between both groups. An untreated group was not included because the study focused at comparing the effect of the SUB+IV combined formulation with any background of the IV immunostimulant alone. The immune markers selected to characterize possible correlates of vaccine protection included IgG antibodies, which have been shown to mediate SUB protective response (Merino et al., 2011, 2013), and the innate immune response-mediated trained immunity markers *akr2, IL-1*β*, IL-2, IL-12, TNF-*α, and *C3* involved in protective response to IV oral or systemic vaccination in different species (Beltrán-Beck et al., 2014; de la Fuente et al., 2016a; Juste et al., 2016; Thomas et al., 2017; López et al., 2018, 2019; Risalde et al., 2018).

### Vaccine Efficacy for the Control of Cattle Tick Infestations

The results of the vaccination trial showed a 51% reduction in the number of engorged female ticks obtained in the SUB+IV group when compared to the IV control (*p* = 0.02; Table 1). This reduction in the number of ticks completing feeding was similar in all three cattle vaccinated with SUB+IV (Table 1). Additionally and despite animal-to-animal variations, a 30% reduction in tick fertility was observed in the eggs from ticks fed on SUB+IV vaccinated calves when compared to the IV control (*p* = 0.04; Table 1). The effect of tick vaccines on the reduction in the number of female ticks completing feeding and fertility has been identified as a critical parameter for the control of cattle tick infestations (Schetters et al., 2016). However, no differences were observed in the weight of engorged ticks or the oviposition (Table 1), a result that differs from previous vaccination trials with systemic SUB (Merino et al., 2011, 2013; Artigas-Jerónimo et al., 2018).

In a previous experiment (Contreras et al., 2015), sera from pigs orally immunized with SUB, SUB-Major surface protein 1a (MSP1a) *E. coli* membrane-bound chimera or PBS as control were used for *R. microplus* capillary feeding to evaluate tick weigh. The results of this preliminary trial showed no effect of anti-SUB antibodies but a 55% reduction in tick weight increase after feeding on SUB-MSP1a serum (Contreras et al., 2015). Bacterial membranes with the surface exposed SUB-MSP1a chimeric antigen were shown to enhance SUB immunogenicity and protective capacity, thus providing supporting evidences for the inclusion of bacterial-derived immunostimulants for SUB-based oral vaccine formulations (Contreras et al., 2015). The discrepancies on the effect of vaccination on tick weight may be attributable to different factors including differences in the immune response to various SUB antigens and between systemic and oral vaccine formulations.

### Correlates of Vaccine Efficacy and Immune Mechanisms of Protection

To address the putative protective mechanisms of oral SUB+IV vaccination in cattle, the IgG antibody response against both SUB and IV (P22) were first characterized in all animals included in the study. The antibody response against SUB has been correlated before with vaccine efficacy in cattle (Merino et al., 2011, 2013). The P22 antigen is composed by various *M. bovis* proteins that have been validated before for the analysis of the antibody response in IV vaccinated hosts (Infantes-Lorenzo et al., 2017, 2018; López et al., 2018; Risalde et al., 2018).

**Figure 2:**
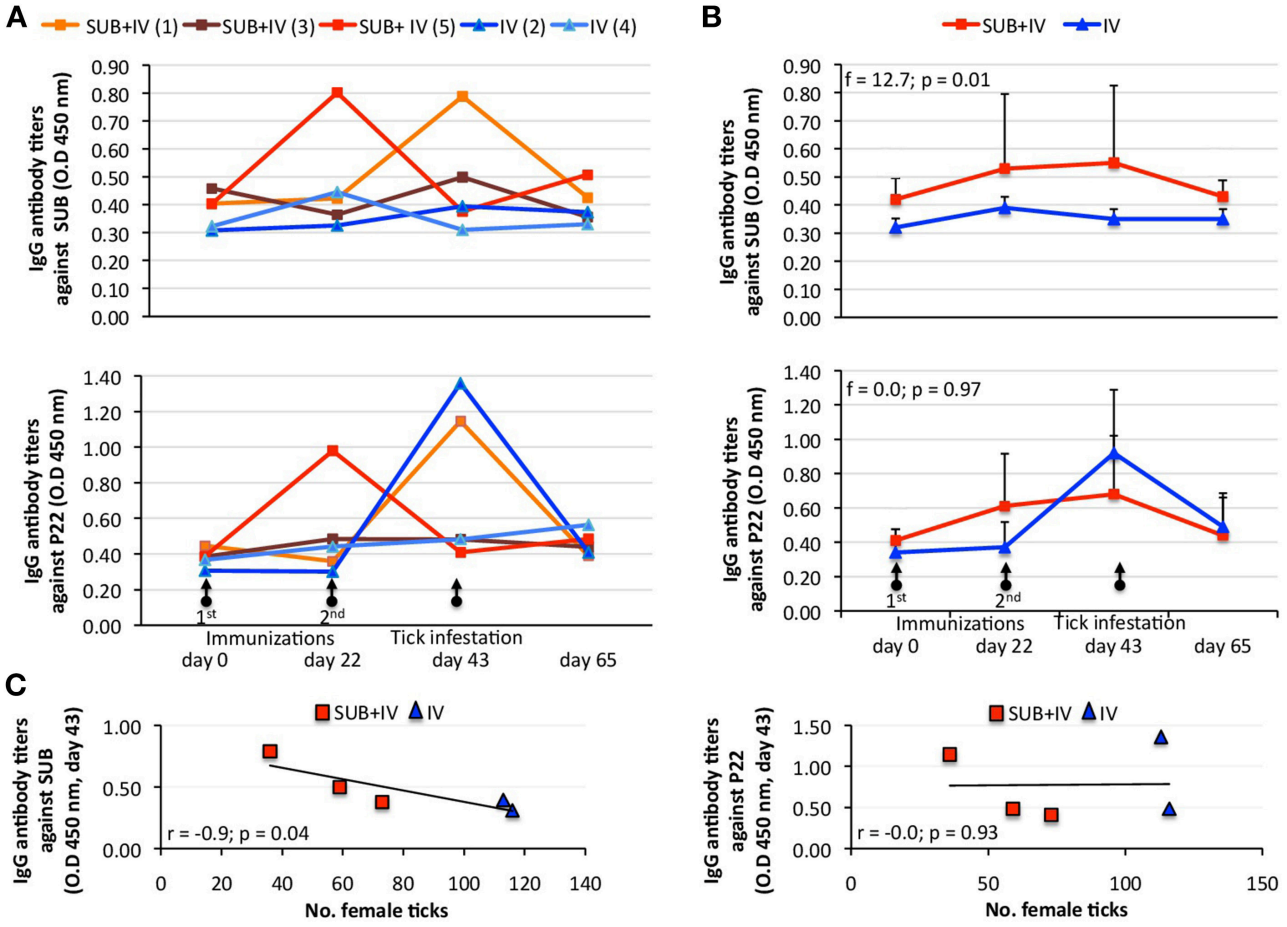
Effect of oral vaccination and infestation with *R. microplus* larvae on the cattle IgG antibody response. Serum samples were collected before 1st (day 0) and 2nd (day 22) immunizations and tick infestation (day 43) with *R. microplus* larvae, and at the end of the experiment (day 65). **(A)** IgG antibody titers were determined by ELISA with serum samples (1:5,000) from individual vaccinated cattle against SUB or P22 proteins. **(B)** Antibody titers in vaccinated cattle were expressed as the average + S.D. O.D_450nm_ (O.D_cattlesera_ – O.D_PBScontrol_) and compared between SUB+IV and IV groups by ANOVA test (*p* < 0.05). **(C)** The Spearman's Rho correlation analyses (*p* < 0.05) were conducted to correlate the IgG antibody response to SUB or P22 at time of tick infestations (day 43) with the vaccine effect on tick. The linear correlation coefficients (r) and *p*-value are shown (*N* = 5).

Despite animal-to-animal differences in the IgG antibody response to both antigens (Figure 2A), the anti-SUB but not anti-P22 IgG antibody response was higher in SUB+IV vaccinated cattle when compared to IV vaccinated group (*p* = 0.01; Figure 2B). Differences in the animal-to-animal response could be attributed to variations in vaccine administration via needleless syringe. Furthermore, a correlation analysis between vaccine efficacy on the reduction in the number of female ticks and anti-SUB or P22 IgG antibody levels was conducted as one of the proposed correlates of protection in tick vaccine trials (de la Fuente and Contreras, 2015). As in previous tick SUB vaccination trials (Merino et al., 2011, 2013), a negative correlation (*r* = −0.9; *p* = 0.04) was obtained between the number of engorged female ticks and IgG antibody titers against SUB but not P22 in vaccinated cattle at day 43 (Figure 2C), showing a correlation between the effect of oral SUB+IV vaccination (IgG antibodies) and vaccine efficacy on tick development.

**Figure 3:**
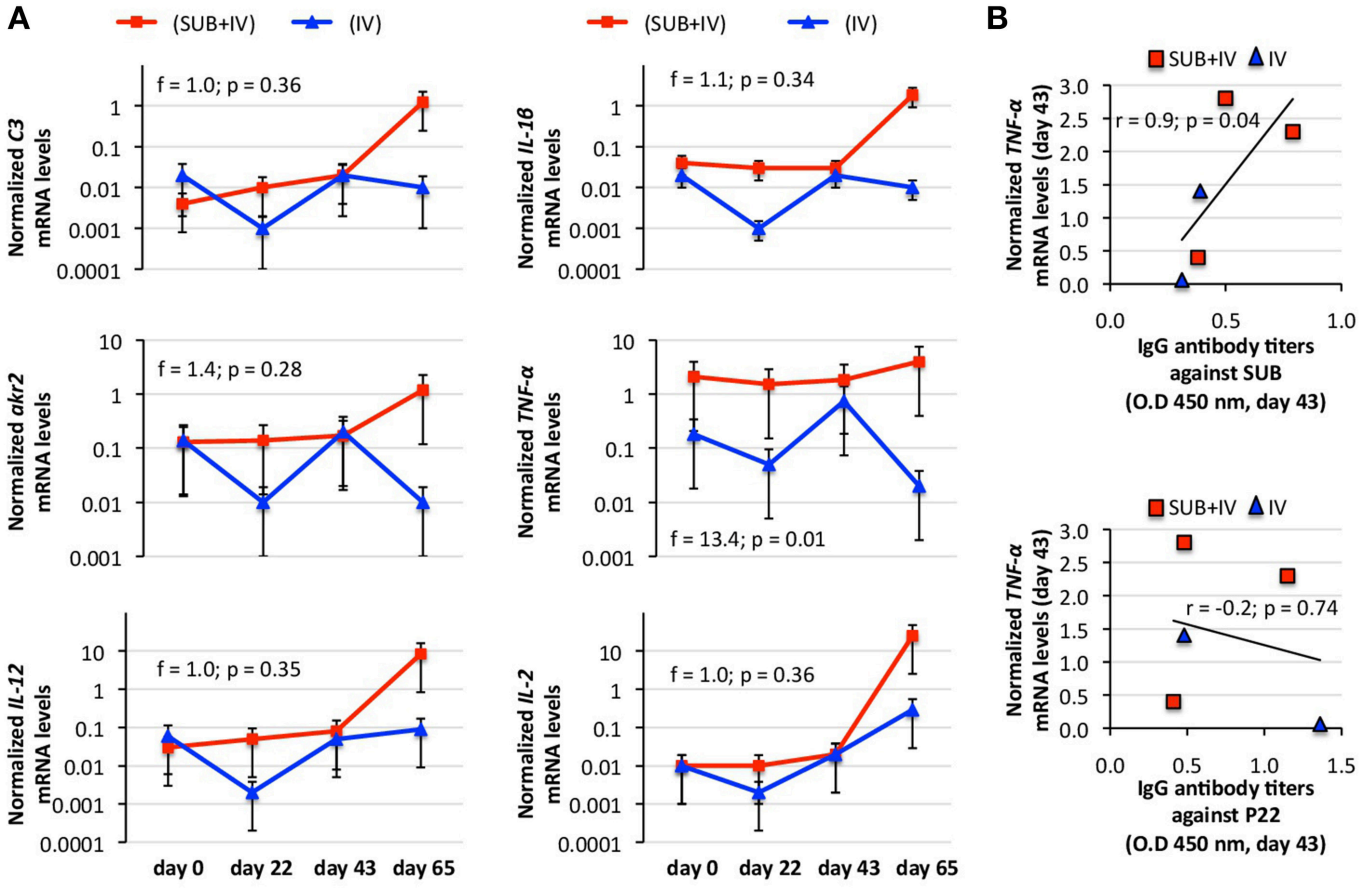
Effect of oral vaccination and infestation with *R. microplus* larvae on the cattle mRNA levels for immune response genes. Blood mRNA was obtained from samples collected before 1st (day 0) and 2nd (day 22) immunizations and tick infestation (day 43) with *R. microplus* larvae, and at the end of the experiment (day 65). **(A)** The mRNA levels for immune response genes *C3, TNF-*α*, IL-1*β, *IL-2, akr2*, and *IL-12* were determined by qRT-PCR in vaccinated cattle, normalized against *B. taurus ß-actin* and normalized mRNA levels (mean of duplicated and normalized Ct values) compared between SUB+IV and IV groups by ANOVA test (*p* < 0.05). **(B)** The Spearman's Rho correlation analyses (*p* < 0.05; https://www.socscistatistics.com/tests/spearman/Default2.aspx) were conducted to correlate the IgG antibody response to mRNA levels at time of tick infestations (day 43). The normalized *TNF-*α mRNA levels were the only that positively correlated with anti-SUB but not P22 IgG antibody levels. The linear correlation coefficients (r) and *p*-value are shown (*N* = 5).

**Figure 4:**
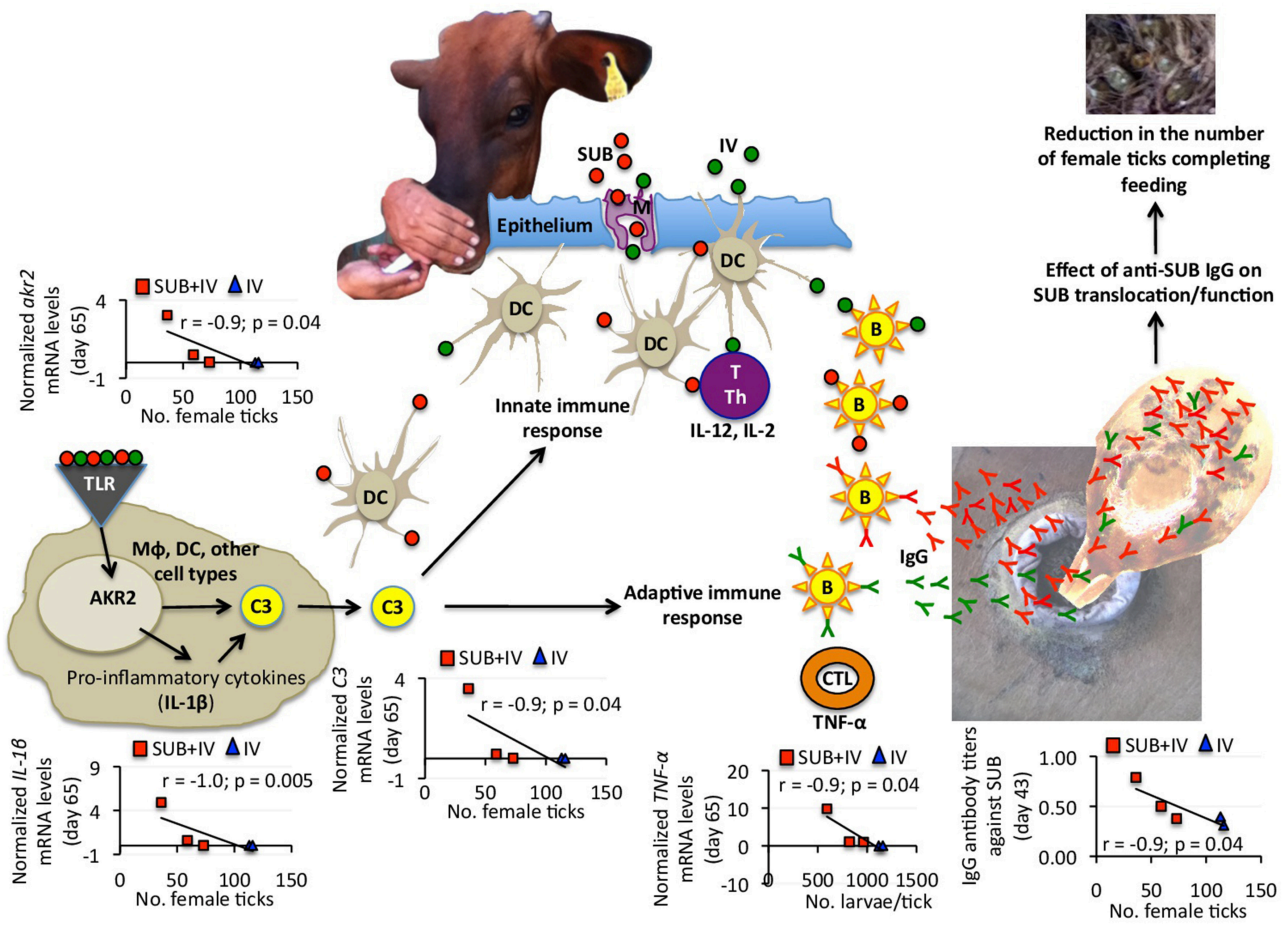
Proposed mechanism of protection for oral SUB+IV vaccine against tick infestations in cattle. Based on the negative correlation between anti-SUB IgG antibody and C3 pathway gene mRNA levels and the number of female ticks, a protective mechanism for SUB+IV oral vaccination was proposed. After the vaccine reaches the oral mucosa-associated lymphoid tissue underlying the epithelium, antigen uptake, processing, and presentation occurs via microfold/membraneous cells (M) by antigen-presenting cells (APCs) such as macrophages (Mϕ), dendritic cells (DCs) and B cells (B). The C3 pathway activated in DCs and other cell types bridge the innate and adaptive immune systems for the initiation of antigen-specific response by activated T cells (T), T helper cells (Th), cytotoxic T lymphocytes (CTLs) and B cells. Activated adaptive immune response cells secrete cytokines such as IL-12, IL-2, and TNF-α to realize the systemic immune protection. The C3 pathway was activated after oral vaccination with SUB+IV to mediate the production of IgG antibodies affecting SUB translocation/function after ingestion with blood meal for the reduction of tick infestations.

The protective mechanism associated with IV vaccination has been attributed to the activation of the innate immune response-mediated trained immunity through C3 pathway (Beltrán-Beck et al., 2014; de la Fuente et al., 2016a; Juste et al., 2016). Therefore, C3 pathway components and other immune response genes were selected for the analysis of mRNA levels in vaccinated cattle. The results showed a tendency toward an increase in mRNA levels in response to vaccination/infestation in SUB+IV vaccinated cattle (Figure 3A). In IV-vaccinated cattle, the tendency was a decrease in mRNA levels at days 22 and 65 following first vaccination and tick infestation, respectively, and an increase at day 43 after second vaccination (Figure 3A). Nevertheless, a positive correlation (*r* = 0.9; *p* = 0.04) was obtained only between *TNF-*α mRNA levels and IgG antibody titers against SUB but not P22 at day 43 (Figure 3B). Additionally, for C3 pathway immune response *akr2, IL-1*β, and *C3* genes a negative correlation (*r* = −0.9 to −1.0; *p* = 0.005 to 0.04) was observed between mRNA levels and the number of engorged female ticks at day 65 (Figure 4). Finally, *TNF-*α mRNA levels negatively correlated (*r* = −0.9; *p* = 0.04) with tick fertility (number of larvae per tick) at day 65 (Figure 4).

Recently, we proposed that the mechanism behind systemic SUB vaccine protective capacity is based on anti-SUB antibodies that could enter into tick cells by still unknown mechanisms where they can interact with cytosolic SUB to prevent its translocation to the nucleus and/or SUB-protein interactions necessary to exert its regulatory functions (de la Fuente et al., 2011) (Figure 4). SUB exerts its function through physical and/or functional interactions with other proteins that have not been fully characterized, but include some candidate proteins involved in the regulation of developmental processes potentially affecting tick feeding (Artigas-Jerónimo et al., 2018). Herein, the IgG antibody response against SUB also correlated with oral vaccination efficacy, therefore suggesting a similar protective mechanism in orally and systemically vaccinated cattle (Figure 4).

Considering the negative correlation between anti-SUB IgG antibody and C3 pathway gene mRNA levels and the number of female ticks, a protective mechanism for SUB+IV oral vaccination was proposed (Figure 4). Once the vaccine reaches the oral mucosa-associated lymphoid tissue underlying the epithelium, antigen uptake, processing and presentation occurs via microfold/membraneous cells (M) by antigen-presenting cells (APCs) such as macrophages (Mϕ), dendritic cells (DCs) and B cells (B) (Wang et al., 2015) (Figure 4). The key APCs, DCs bridge the innate and adaptive immune systems for the initiation of antigen-specific response by activated T cells (T), T helper cells (Th), cytotoxic T lymphocytes (CTLs) and B cells (Holmgren and Czerkinsky, 2005; Peng et al., 2006; Lawson et al., 2011; Wang et al., 2015) (Figure 4). The C3 pathway mediates bridging of the innate and adaptive immune responses (Lubbers et al., 2017) (Figure 4). Activated adaptive immune response cells secrete cytokines such as IL-12, IL-2, and TNF-α, which mRNA levels positively correlated with anti-SUB antibody titers (Figure 3B), and then migrate to and multiply through the common mucosal immune system to realize the systemic immune protection (Seder and Hill, 2000; Wang et al., 2015). As also shown in this study, the production of neutralizing antibodies is vital for protective humoral immune response in reducing tick infestations (de la Fuente et al., 1998, 2011; Merino et al., 2011, 2013; Contreras and de la Fuente, 2016, 2017). In this process, the C3 pathway that was activated after oral vaccination with SUB+IV has been shown to mediate antibody response (Rutemark et al., 2011), a finding supported here by the positive correlation between *akr2, IL-1*β, and *C3* mRNA levels and the number of female ticks (Figure 4). Additionally, the proinflammatory cytokine IL-1β has been reported to induce antigen-specific serum IgG and lymphocyte proliferative responses with intranasally administered with soluble protein antigens (Staats and Ennis, 1999).

Finally, the negative correlation between *TNF-*α mRNA levels and the number of larvae per tick suggested that T-mediated response after SUB+IV oral vaccination may have a role in the reduction of tick fertility. As discussed above and supported in this study, antigen-specific IgG antibody response plays a key role in the protective response to tick vaccines (de la Fuente et al., 1998, 2011; Merino et al., 2011, 2013; Contreras and de la Fuente, 2016, 2017). However, the possible role of the complement, T immune response and other effector mechanisms has been considered before as part of tick vaccine efficacy (Tellam et al., 1992). The development of specific antibody- or T-mediated immunologic responses and the activation of mucosally-induced immunity depend on complex sets of immunologic events, including the antigen-induced activation of different populations of B, T, and APC, and the expression of proinflammatory and immunoregulatory cytokines (Dinarello, 2000; Ogra et al., 2001). In particular, the proinflammatory cytokine TNF-α has been associated with DC maturation in mucosal immune response (Ogra et al., 2001). The results obtained here suggested that future experiments should address this component of the protective immune response to both systemic and oral vaccination with tick antigens. Additionally, other events and cytokines such as IL-4, IL-5, IL-6, IL-10, colony-stimulating factor (CSF), interferon gamma (IFN-γ) and transforming growth factor beta (TGF-β) reported to be involved in mucosal immune responses together with variations in APC, B, and T cell subsets should be addressed to provide additional support for the proposed protective mechanisms elicited by oral vaccination with SUB+IV.

## Conclusions

The results of this study confirmed the efficacy of tick SUB for the control of cattle tick infestations, and expanded these results to oral vaccination with this antigen in combination with the IV immunostimulant. Furthermore, the identification of correlates of oral SUB+IV vaccine protection provided markers and targets for future experiments with larger number of animals aimed at conducting analyses to provide additional support for the protective immune mechanisms, the duration of the protective response, and optimizing vaccine formulation, delivery and efficacy. An optimal oral vaccine formulation requires the appropriate combination of antigens, immunostimulants and delivery carriers to induce a series of protective immune responses as shown here by the activation of the C3 pathway and the production of IgG antibodies and relevant cytokines (Figure 4). Research focused on oral tick vaccines would have a positive impact on the control of tick infestations and tick-borne diseases in humans, farm and companion animals through reducing stress and systemic vaccination-associated risks while increasing protective efficacy.

## Data Availability

All datasets generated for this study are included in the manuscript and/or the supplementary files.

## Ethics Statement

The study was conducted in accordance with standards specified in the Guide for Care and Use of Laboratory Animals for the University of Tamaulipas (UAT), Mexico. The protocol was approved by the ethics committee of the UAT (No. CBBA_17_0).

## Author Contributions

MC, OM, CG, and JF conceived the study and designed the experiments. MC, PK, OM, and NdlC-H performed the experiments. MC and JF performed data analysis. MC, PK, CG, and JF wrote the manuscript. All authors approved and contributed to the final version of the manuscript.

### Conflict of Interest Statement

The authors declare that the research was conducted in the absence of any commercial or financial relationships that could be construed as a potential conflict of interest.
